# Diagnostic efficacy of serum ST2 in patients with ASC

**DOI:** 10.1002/jcla.24511

**Published:** 2022-05-25

**Authors:** Yaping Ren, Min Hou, Yunxia Ren, Lei Zhang

**Affiliations:** ^1^ Department of Cardiology, Shanxi Bethune Hospital, Shanxi Academy of Medical Sciences, Tongji Shanxi Hospital Third Hospital of Shanxi Medical University Taiyuan China; ^2^ Emergency Department, Shanxi Bethune Hospital, Shanxi Academy of Medical Sciences, Tongji Shanxi Hospital Third Hospital of Shanxi Medical University Taiyuan China

**Keywords:** acute coronary syndrome, cutoff value, non‐ST‐segment elevation myocardial infarction, receiver‐operating characteristic curves, soluble suppression of tumorigenicity 2

## Abstract

**Background:**

Soluble suppression of tumorigenicity 2 (ST2) is closely related to the development of cardiovascular disease, but the level of acute coronary syndrome (ACS) and the relationship between ST2 and ACS are unclear.

**Patients and Methods:**

Patients with the acute coronary syndrome were divided into the unstable angina pectoris (USAP) group (*n* = 65) and non‐ST‐segment elevation myocardial infarction (NSTEMI) group (*n* = 58), and the healthy population, without chest pain and with normal coronary CT, was included as a control group (*n* = 55). Laboratory index levels were collected from each participant. The baseline information was reviewed and analyzed. The binary logistic regression was used to explore the relation of ST2 levels with the occurrence of ACS and NSTEMI, and the diagnostic performance of ST2 for diagnosing ACS or NSTEMI was evaluated using a receiver‐operating characteristic (ROC) curve.

**Results:**

The level of ST2 was found significantly higher in NSTEMI than in USAP and was higher in USAP than in control (*p* < 0.01). ST2 levels were positively correlated with ALT, AST, and BNP in the control group, were negatively correlated with HGB and TG in the USAP group, and were positively correlated with WBC, GLU, BNP, and Gensini scores in the NSTEMI group. Multivariate analysis revealed that the occurrence of ACS was associated with ST2, BNP, GLU, TC, BUN, WBC, and PLT, and the occurrence of NSTEMI was associated with AST, WBC, LDL‐C, and ST2. Meanwhile, ST2 levels achieved good performance for ACS and NSTEMI diagnostician.

**Conclusion:**

ST2 could be used as an auxiliary diagnostic indicator for the occurrence of ACS and NSTEMI.

## Graphical abstract

The diagnostic performance of ST2 for ACS was equivalent to BNP, and the diagnostic performance of ST2 for NSTEMI was equivalent to AST and WBC. ST2 could be used as an auxiliary diagnostic indicator for the occurrence of ACS and NSTEMI.

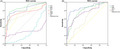

## 1 INTRODUCTION

Acute coronary syndrome (ACS) is a clinical syndrome based on the pathology of ruptured or invasive coronary atherosclerotic plaques secondary to complete or incomplete occlusive thrombosis, including acute ST‐segment elevation myocardial infarction, acute non‐ST‐segment elevation myocardial infarction, and unstable angina pectoris (USAP). Despite a variety of therapeutic measures such as emergency thrombolytic therapy, percutaneous coronary stenting (PCI) and coronary artery bypass grafting are now widely used in clinical practice, the risk of major adverse cardiovascular events (MACEs) in patients with acute myocardial infarction (AMI) remains increasing.^1^

The clinical presentation of ACS is typical in only a few cases.^2^ Therefore, timely diagnosis is essential for the selection of appropriate evidence‐based therapies, while the accurate and effective exclusion of AMI helps to avoid human damage and efficient use of medical resources.^3^

Clinical features and electrocardiography provide timely recognition of ST‐segment elevation myocardial infarction (STEMI) and are considered to be the main criteria for the diagnosis of AMI, but not sufficient to diagnose or exclude NSTEMI in the majority of patients.^4^ Although STE of aVR (≥1 mm) has been commonly used as a marker of LM infarction, it has been reported^5^ that there may still be 20% to 38% of patients with acute total LM occlusion without aVR ST‐segment elevation. The Gensini score is a common method for assessing the extent of coronary artery disease in clinical practice. Studies have confirmed the good value of this score in assessing the condition of patients with coronary artery disease.^6^ The Grace and TIMI scores are well‐known scores and supported by current clinical guidelines, but seem to be more appropriate as a (short‐term) prognostic score for patients already diagnosed with ACS.^7^, ^8^ Currently, high‐sensitivity cardiac troponin T (hs‐cTnT) or high‐sensitivity cardiac troponin I (hs‐cTnI) is complementary to clinical assessment as a key indicator in the initial assessment of NSTEMI,^9^ but is susceptible to the influence of other diseases, which in turn affects the accuracy of NSTEMI diagnosis.^10^, ^11^

Soluble suppression of tumorigenicity 2 (ST2) is a member of the interleukin 1 receptor family, formally known as interleukin 1 receptor‐like 1 (IL1RL‐1). There are two major isoforms: a transmembrane receptor (ST2L) and a truncated soluble receptor, which can be detected in the serum.^12^ The interaction between interleukin (IL)‐33 and ST2L is upregulated during myocardial stress and protects the myocardium by reducing fibrosis and hypertrophy, and improving survival.^13^ The soluble form of ST2 acts as a decoy receptor that inhibits the cardioprotective effects of IL‐33, leading to cardiac hypertrophy, myocardial fibrosis, and ventricular dysfunction, and be used to assess the severity and prognosis of heart failure.^14^ Additionally, Demyanets et al. reported that serum sST2 levels were significantly higher in patients with ACS compared with patients with stable coronary artery disease and without coronary artery disease, and were similar between the two groups in the stable angina and control groups.^15^ However, only a limited number of studies have investigated the performance of sST2 levels in ACS. In this study, we assessed the levels and diagnostic value of sST2 in ACS. We hypothesized that sST2 is associated with the development of ACS.

## 2 MATERIALS AND METHODS

### 2.1 Subjects

The study was reviewed and approved by the Ethics committee of Shanxi Baiqiuen Hospital. Signed informed consent was obtained from the participants before enrollment in the study. 123 patients with ACS (ACS group) (78 men; mean age, 58.65 ± 10.87 years old) and 55 health screeners with the normal coronary artery (control group) (28 men; mean age, 57.93 ± 7.37 years old) were included in this study. ACS was divided into two subgroups: USAP, which was defined as a patient with symptoms of myocardial ischemia but no increase in troponin, with or without ischemic changes in the electrocardiogram, such as ST‐segment depression or new T wave inversion, and included 65 patients. NSTEMI was determined as a patient with symptoms of myocardial ischemia and increased in troponin, with ischemic changes in the electrocardiogram, included 58 patients, and all NSTEMI patients were Killip class I. All patients with ACS presented with typical ischemic chest pain within 6 h of symptom onset, without ST‐segment elevation on ECG, and underwent diagnostic coronary angiography. Controls were evaluated by electrocardiogram, echocardiogram, and coronary CT, which showed no abnormalities. The exclusion criteria for all participants were as follows: i) Participants with infections, tumors, or systemic immune diseases; ii) who with severe liver and kidney diseases; iii) patients with severe hypertension with uncontrolled blood pressure, and diabetes mellitus with long‐term uncontrolled blood sugar within the ideal range; iv) who with more than moderate valvular disease, heart failure, hypertrophic cardiomyopathy, thyroid disease, severe anemia; v) patients use of immunosuppressive drugs.

### 2.2 Clinical and biochemical analysis

Participants were asked to complete a questionnaire on medical history, medication use, height, and weight. All participants underwent chest radiographs, abdominal ultrasonography, electrocardiography, and echocardiography. Left atrial diameter (LAd) and left ventricular end‐diastolic dimension (LVEDD) were measured, and left ventricular ejection fraction (LVEF) was calculated by the Simpson's biplane method.

Coronary angiography was performed in all patients with ACS, and the degree of stenosis in the left main coronary artery, the left anterior descending branch, the left rotating branch, and the right coronary artery was assessed in conjunction with the imaging findings. A lesion was considered normal if there was no abnormality or if the stenosis was <50%; a single‐branch lesion was determined if ≥50% stenosis was present in any of the above arteries, and a multi‐branch lesion was determined if ≥50% stenosis was present in two or three arteries. According to the American Heart Association's Gensini score, stenosis ≤25% was scored as 1, 26%–50% as 2, 51%–75% as 4, 76%–90% as 8, stenosis 91%–99% as 16, and complete occlusion as 32, and the final Gensini score was obtained by multiplying the stenosis score of the artery being evaluated by the correlation coefficient. The GRACE score is a tool that can assess the risk and prognosis of ischemic adverse events in patients with CHD, including age, systolic blood pressure, pulse rate, and blood creatinine. The sum of the scores of each index is the GRACE score. 0–109 is considered low risk, 110–140 is considered the intermediate risk, and 141 and above is considered high risk. The TIMI risk score is a simple prediction scheme to classify the risk of death and ischemic events in patients with USAP/NSTEMI.^16^

Venous blood is usually collected within 6 h of admission, prior to angiography and administration of medication, and centrifuged at 3000 rpm for 10 min at 4°C to separate plasma and serum. Plasma and serum were aliquoted in Eppendorf tubes and frozen at −80°C until analysis. ST2 was measured using an ST2 assay kit (R&D Systems, USA). The levels of alanine transaminase (ALT), aspartate aminotransferase (AST), blood urea nitrogen (BUN), serum creatinine (sCr), uric acid (UA), blood glucose (GLU), total cholesterol (TC), low‐density lipoprotein cholesterol (LDL‐C), triglyceride (TG), and brain natriuretic peptide (BNP) were determined using the automatic biochemical analyzer (Hitachi, Tokyo, Japan). White blood cells (WBC), hemoglobin (HGB), and platelet (PLT) were measured by an automatic blood cell analyzer (Beckman Coulter, Miami, FL, USA).

### 2.3 Statistical analysis

SPSS 22.0 software (IBM Corp.) was used for the statistical analyses. T‐test or Mann–Whitney *U* test was used for the intergroup comparisons. Mean ± standard deviation (SD) or median (quartiles) was used for the presentation of the results. Chi‐square (*χ* ^2^) tests were used for ratio comparisons intergroup. Fisher exact tests were used when the expected count was <5. The Pearson's or Spearman correlation analysis was used to confirm the association between the variables. The binary logistic regression analyses were used to analyze the relationship between the ST2 levels and other important factors associated with the ACS or NSTEMI (*p* ≤ 0.05) in the multivariate analyses using stepwise selection. Receiver Operating Characteristics (ROC) curves and area under ROC (AUROC) were used to predict the occurrence of ACS or NSTEMI. The cutoff value of ST2 was determined by the nearest integer (Youden index). The two‐tailed test was set at a significance level of 0.05.

### 2.4 Sample size calculation

The sample size was calculated using G power version 3.0.10. The minimum sample size of patients needed to get a power level of 0.80, an alpha level of 0.05, and a medium effect size of 0.50 for ST2 was 52 in each group.

## 3 RESULTS

### 3.1 Clinical characteristics

The characteristics of the participants were shown in Table 1. No significant differences were found in age, sex, ECG parameters, and most of the comorbidities between ACS and controls. However, compared with controls, the levels of BMI, systolic pressure, diastolic pressure, LAd, LVEDD, and patients with hypertension, diabetes, and heart shadow were significantly higher, LVEF was significantly lower in ACS (*p* < 0.05 or *p* < 0.01). The levels of LVEDD in the NSTEMI subgroup were much higher (*p* < 0.05), and the level of LVEF was notable lower (*p* < 0.01) compared with the USAP subgroup, and there were more males than females in the NSTEMI subgroup (*p* < 0.05). Levels of Grace scores, TIMI scores, and Gensini scores were much higher than the USAP subgroup (*p* < 0.01), and most of the ECG parameters were significantly different in the UA and NST groups (*p* < 0.05 or *p* < 0.01). (Table 1).

**TABLE 1:** Characteristics of subjects

	Controls (*N* = 55)	ACS (*N* = 123)	USAP (*N* = 65)	NSTEMI (*N* = 58)
Age (years)	57.93 ± 7.37	58.65 ± 10.87	60.42 ± 9.60	56.67 ± 11.92
Male/female	28/27	78/45	34/31	44/14^#^
BMI (kg/m^2^)	22.15 ± 1.79	25.14 ± 3.01^**^	25.35 ± 2.97	25.18 ± 3.43
systolic pressure	125.51 ± 8.16	131.78 ± 17.62^**^	131.92 ± 16.89	131.62 ± 18.56
diastolic pressure	74.33 ± 5.29	79.92 ± 12.48^**^	80.0 (70.0, 88.0)	79.0 (72.0, 86.0)
LAd (mm)	32.36 ± 1.71	33.50 ± 3.26^**^	33.0 (32.0, 35.0)	34.0 (32.0, 35.0)
LVEDD (mm)	46.27 ± 1.92	48.33 ± 5.60^**^	47.0 (44.0, 50.0)	48.5 (48.0, 50.0)^#^
LVEF (%)	64.80 ± 3.03	58.40 ± 8.48^**^	63.0 (59.5, 66.0)	54.0 (58.0, 62.0)^##^
Hypertension	6	63^*^	29	34
Diabetes	0	21^**^	13	9
Gastric ulcer/gastritis	0	2	1	1
Hyperthyroidism/hypothyroidism	0	2	1	1
Heart shadow	0	10^*^	3	7
Fatty liver	2	15	8	7
Anemia	0	1	1	0
Increased lung texture	2	11	8	3
ST2 (ng/ml)	3.8 (0.0, 5.0)	16.0 (6.3, 28.9)^**^	9.40 (3.80, 17.40)	23.45 (12.40, 55.60)^**^ ^,^ ^#^
cTnI (μg/L)	0.02 (0.00, 0.04)	0.30 (0.19, 3.76)^**^	0.25 (0.12, 0.30)	4.50 (1.26, 12.30)^**^ ^,^ ^##^
BUN (mmol/L)	4.10 (3.80, 4.95)	5.10 (4.40, 6.40)^**^	5.3 (4.5, 6.7)^**^	5.0 (4.1, 6.3)^**^
AST(U/L)	24.5 (18.0, 32.0)	22.7 (18.5, 45.0)	19.3 (16.3, 22.3)	45.0 (28.1, 68.0)^**^ ^,^ ^##^
ALT(U/L)	16.0 (13.0, 21.0)	21.0 (16.0, 32.0)^**^	17.60 (14.35, 22.60)	28.10 (19.30, 41.80)^**^ ^,^ ^##^
sCr (μmol/L)	78.0 (71.2, 87.6)	78.0 (70.3, 88.8)	74.30 (65.45, 84.40)	81.80 (75.20, 92.00)^**^ ^,^ ^##^
UA (μmol/L)	312.0 (264.0, 342.5)	325.0 (275.6, 389.3)	327.56 ± 100.32	345.50 ± 99.23
WBC (×10^9^/L)	5.3 (5.1, 5.6)	6.60 (5.3, 8.2)^**^	5.8 (4.8, 6.9)	7.9 (6.5, 9.0)^**^ ^,^ ^##^
HGB (g/L)	134.0 (126.0, 138.0)	137.0 (126.0, 148.0)^*^	140.0 (126.5, 148.5)	136.5 (125.0, 148.0)
PLT (×10^9^/L)	246.0 (211.5, 318.0)	203.0 (168.0, 234.0)^**^	200.55 ± 54.28	212.24 ± 56.19
GLU (mmol/L)	5.10 (4.75, 5.30)	5.51 (5.02, 6.32)^**^	5.35 (5.01, 6.17)	5.73 (5.12, 6.40)
TC (mmol/L)	4.90 (4.80, 5.15)	4.19 (3.60, 5.10)^**^	4.14 (3.20, 4.74)	4.30 (3.87, 5.19)
LDL‐C (mmol/L)	2.70 (2.10, 2.90)	2.47 (1.88, 3.00)	2.35 (1.61, 2.88)	2.57 (2.24, 3.30)^*^ ^,^ ^#^
TG (mmol/L)	1.78 (1.66, 1.86)	1.54 (1.04, 2.20)^*^	1.38 (1.04, 2.13)	1.66 (1.04, 2.58)
BNP(ng/L)	23.0 (11.5, 33.0)	80.0 (40.0, 235.0)^**^	52.5 (30.0, 79.0)	196.5 (95.0, 507.0)^**^ ^,^ ^##^
Grace scores	——	105.48 ± 28.53	93.82 ± 19.84	133.56 ± 5.17^##^
TIMI scores	——	2.93 ± 1.64	2.37 ± 1.14	3.55 ± 1.88^##^
Gensini scores	——	35.36 ± 31.77	15.01 ± 19.84	58.16 ± 26.78^##^
heart rate	71.5 ± 10.91	70.16 ± 10.50	70.43 ± 9.95	69.86 ± 11.17
PR interval	0.14 ± 0.01	0.14 ± 0.04	0.17 ± 0.02^**^	0.11 ± 0.02^##^
P wave duration	0.09 ± 0.01	0.10 ± 0.02	0.10 ± 0.01^*^	0.09 ± 0.03^#^
P wave amplitude	0.10 ± 0.04	0.13 ± 0.04	0.10 ± 0.02	0.16 ± 0.03^##^
QRS complex	0.09 ± 0.01	0.10 ± 0.09	0.11 ± 0.12	0.09 ± 0.02
QTc interval	0.38 ± 0.04	0.40 ± 0.05	0.39 ± 0.03	0.41 ± 0.06^#^
QTc duration	0.40 ± 0.04	0.43 ± 0.05	0.42 ± 0.02	0.44 ± 0.06^#^

Abbreviations: ACS, Acute acute coronary syndrome; ALT, alanine transaminase; AST, aspartate aminotransferase; BMI, body mass index; BNP, brain natriuretic peptide; BUN, blood urea nitrogen; cTnI, cardiac troponin I; GLU, blood glucose; HGB, hemoglobin; LAd, left atrial diameter; LDL‐C, low‐ density lipoprotein cholesterol; LVEDD, left ventricular end‐diastolic dimension; LVEF, left ventricular ejection fraction; NSTEMI, non‐ST‐ segment elevation myocardial infarction; PLT, platelet count; sCr, serum creatinine; ST2, suppression of tumorigenicity 2; TC, total cholesterol; TG, triglyceride; UA, uric acid; USAP, unstable angina pectoris; WBC, white blood cells.

Bold values represent the correlations reached statistically significant.

^*^ *p* < 0.05 versus controls.

^**^ *p* < 0.01 versus control.

^#^ *p* < 0.05 versus UAP subgroup.

^##^ *p* < 0.01 versus UAP subgroup.

### 3.2 Serum levels of laboratory indicators

The levels of ST2, cTnI, BUN, ALT, WBC, HGB, GLU, and BNP in the ACS group were notably higher than in the controls, and the levels of PLT, TC, and TG were significantly lower than in the control group (*p* < 0.01). The concentrations of ST2, cTnI, ALT, AST, sCr, WBC, LDL‐C, and BNP in the NSTEMI subgroup were much higher than in the USAP subgroup (*p* < 0.01 or *p* < 0.05). (Table 1).

### 3.3 Relationship between ST2 and other indicators

We performed a correlation analysis of laboratory indicators and ST2 in controls, USAP, and NSTEMI. Spearman correlation analysis showed positive correlations between ST2 and ALT (*r* = 0.299, *p* = 0.027), AST (*r* = 0.288, *p* = 0.033), and BNP (*r* = 0.297, *p* = 0.028) in the control group. And there were negative correlations between ST2 and HGB (*r* = −0.278, *p* = 0.018), TG (*r* = −0.254, *p* = 0.032) in USAP. For NSTEMI, there were correlations between ST2 and WBC (*r* = 0.281, *p* = 0.033), GLU (*r* = 0.366, *p* = 0.005), BNP (*r* = 0.429, *p* = 0.001), and Gensini score (*r* = 0.277, *p* = 0.035) (Table 2). Furthermore, these above‐mentioned variables were incorporated into a multivariate logistic regression model and subsequently, it showed that serum ST2 levels (OR = 1.166; 95% CI, 1.049–1.297, *p* < 0.01), BNP (OR = 1.027; 95% CI, 1.005–1.050, *p* < 0.05), GLU (OR = 9.802; 95% CI, 2.059–46.656, *p* < 0.01), TC (OR = 0.481; 95% CI, 0.253–0.917, *p* < 0.05), BUN (OR = 2.148; 95% CI, 1.158–3.985, *p* < 0.05), WBC (OR = 2.302; 95% CI, 1.213–4.370, *p* < 0.05), and PLT (OR = 0.975; 95% CI, 0.959–0.992, *p* < 0.05) were the independent factors for the development of ACS (Table 3). And serum ST2 levels (OR = 1.035; 95% CI, 1.006–1.066, *p* < 0.05), AST (OR = 1.109; 95% CI, 1.046–1.177, *p* < 0.01), WBC (OR = 1.649; 95% CI, 1.111–2.447, *p* < 0.05), and LDL‐C (OR = 1.851; 95% CI, 1.006–3.405, *p* < 0.05) were the independent factors for the development of NSTEMI (Table 4). ST2 remained an independent factor in the occurrence of ACS or NSTEMI, even after adjusting for age, sex, or BMI.

**TABLE 2:** Relationships between laboratory indicators and ST2

Laboratory indicators	ST2					
	Control		USAP		NSTEMI	
	*r*	*p*	*r*	*p*	*r*	*p*
ALT (U/L)	0.299	**0.027**	−0.210	0.077	0.043	0.751
AST (U/L)	0.288	**0.033**	−0.218	0.066	0.152	0.254
WBC (×10^9^/L)	0.021	0.882	0.123	0.304	0.281	**0.033**
HGB (g/L)	0.158	0.249	−0.278	**0.018**	−0.045	0.739
GLU (mmol/L)	0.029	0.834	−0.015	0.706	0.366	**0.005**
TG (mmol/L)	−0.044	0.749	−0.254	**0.032**	−0.170	0.203
BNP (ng/L)	0.297	**0.028**	0.142	0.242	0.429	**0.001**
cTnI (μg/L)	0.130	0.345	0.155	0.219	0.099	0.630
Grace scores	–	–	0.027	0.829	0.277	0.161
TIMI scores	–	–	−0.131	0.297	−0.093	0.486
Gensini scores	–	–	0.076	0.548	0.277	**0.035**
heart rate	0.243	0.757	0.129	0.304	0.114	0.392
PR interval	−0.472	0.146	−0.096	0.445	0.015	0.911
P wave duration	0.754	0.158	0.205	0.102	0.017	0.896
P wave amplitude	−0.011	0.668	0.133	0.289	−0.071	0.597
QRS complex	0.652	0.348	−0.059	0.640	0.100	0.455
QTc interval	0.006	0.854	−0.230	0.065	0.002	0.988
QTc duration	0.011	0.532	−0.180	0.152	0.083	0.538

Abbreviations: ALT, alanine transaminase; AST, aspartate aminotransferase; BNP, brain natriuretic peptide; cTnI, cardiac troponin I; Glu, blood glucose; HGB, hemoglobin; NSTEMI, non‐ST‐segment elevation myocardial infarction; ST2, suppression of tumorigenicity 2; TG, triglyceride; USAP, unstable angina pectoris; WBC, white blood cells.

Bold values represent the correlations reached statistically significant.

**TABLE 3:** Multivariate logistic regression analysis of the occurrence of ACS

	Multivariate analysis unadjusted		Multivariate analysis adjusted for age		Multivariate analysis adjusted for gender		Multivariate analysis adjusted for BMI		Multivariate analysis adjusted for age, gender, and BMI	
	OR 95% CI	*p*‐value	OR 95% CI	*p*‐value	OR 95% CI	*p*‐value	OR 95% CI	*p*‐value	OR 95% CI	*p*‐value
AST (U/L)	0.991 (0.957–1.025)	0.589	0.982 (0.946–1.019)	0.342	0.999 (0.963–1.037)	0.977	0.982 (0.942–1.024)	0.394	0.979 (0.933–1.028)	0.395
ST2 (ng/ml)	1.166 (1.049–1.297)	**0.005**	1.166 (1.042–1.305)	**0.008**	1.158 (1.045–1.284)	**0.005**	1.165 (1.026–1.322)	**0.018**	1.166 (1.018–1.335)	**0.027**
BNP (ng/L)	1.027 (1.005–1.050)	**0.017**	1.032 (1.007–1.057)	**0.012**	1.028 (1.005–1.053)	**0.019**	1.028 (1.004–1.051)	**0.019**	1.034 (1.007–1.063)	**0.014**
GLU (mmol/L)	9.802 (2.059–46.656)	**0.004**	11.970 (2.451–58.455)	**0.002**	12.368 (2.314–66.104)	**0.003**	9.822 (1.892–50.990)	**0.007**	14.911 (2.452–90.656)	**0.003**
TC (mmol/L)	0.481 (0.253–0.917)	**0.026**	0.443 (0.224–0.879)	**0.020**	0.435 (0.222–0.852)	**0.015**	0.423 (0.209–0.854)	**0.016**	0.334 (0.145–0.769)	**0.010**
BUN (mmol/L)	2.148 (1.158–3.985)	**0.015**	2.239 (1.185–4.234)	**0.013**	2.168 (1.157–4.063)	**0.016**	2.167 (1.070–4.387)	**0.032**	2.411 (1.109–5.241)	**0.026**
WBC (×10^9^/L)	2.302 (1.213–4.370)	**0.011**	2.644 (1.279–5.465)	**0.009**	2.569 (1.312–5.031)	**0.006**	1.902 (0.981–3.690)	0.057	2.418 (1.056–5.537)	**0.037**
PLT (×10^9^/L)	0.975 (0.959–0.992)	**0.004**	0.975 (0.958–0.992)	**0.005**	0.974 (0.957–0.992)	**0.004**	0.979 (0.960–0.998)	**0.034**	0.978 (0.957–1.000)	**0.047**
Age (years)	–	–	0.946 (0.864–1.037)	0.236	–	–	–	–	0.935 (0.844–1.035)	0.196
Gender	–	–	–	–	4.216 (0.806–22.053)	0.088	–	–	2.978 (0.518–17.132)	0.222
BMI (kg/m^2^)	–	–	–	–	–	–	1.567 (1.131–2.171)	**0.007**	1.554 (1.091–2.212)	**0.015**

Abbreviations: AST, aspartate aminotransferase; BMI, body mass index; BNP, brain natriuretic peptide; BUN, blood urea nitrogen; GLU, blood glucose; PLT, platelet; ST2, suppression of tumorigenicity 2; TC, total cholesterol; WBC, white blood cells.

Bold values represent the correlations reached statistically significant.

**TABLE 4:** Multivariate logistic regression analysis of the occurrence of NSTEMI in patients with ACS

	Multivariate analysis unadjusted		Multivariate analysis adjusted for age		Multivariate analysis adjusted for gender		Multivariate analysis adjusted for BMI		Multivariate analysis adjusted for age, gender, and BMI	
	OR 95% CI	*p*‐value	OR 95% CI	*p*‐value	OR 95% CI	*p*‐value	OR 95% CI	*p*‐value	OR 95% CI	*p*‐value
ALT (U/L)	1.021 (0.966–1.059)	0.622	1.013 (0.963–1.066)	0.617	1.010 (0.962–1.059)	0.696	1.014 (0.969–1.062)	0.540	1.017 (0.966–1.071)	0.528
AST (U/L)	1.109 (1.046–1.177)	**0.001**	1.113 (1.049–1.180)	**<0.001**	1.111 (1.046–1.180)	**0.001**	1.101 (1.035–1.172)	**0.002**	1.101 (1.035–1.172)	**0.002**
ST2(ng/mL)	1.035 (1.006–1.066)	**0.018**	1.034 (1.006–1.063)	**0.019**	1.036 (1.006–1.066)	**0.018**	1.036 (1.006–1.066)	**0.019**	1.034 (1.005–1.064)	**0.019**
sCr (μmol/L)	1.005 (1.000–1.011)	0.059	1.006 (1.000–1.012)	0.064	1.005 (1.000–1.011)	**0.049**	1.006 (1.000–1.012)	0.051	1.006 (1.000–1.012)	**0.034**
WBC (×10^9^/L)	1.649 (1.111–2.447)	**0.013**	1.703 (1.140–2.545)	**0.009**	1.650 (1.116–2.440)	**0.012**	1.644 (1.105–2.447)	**0.014**	1.702 (1.134–2.555)	**0.010**
LDL‐C (mmol/L)	1.851 (1.006–3.405)	**0.048**	1.725 (0.933–3.189)	0.082	2.025 (1.072–3.827)	**0.030**	1.856 (1.016–3.391)	**0.044**	1.880 (1.001–3.532)	0.050
Age (years)	–	–	0.956 (0.903–1.012)	0.124	–	–	–	–	0.960 (0.901–1.024)	0.213
Gender	–	–	–	–	0.361 (0.101–1.287)	0.116	–	–	0.422 (0.106–1.682)	0.222
BMI (kg/m^2^)	–	–	–	–	–	–	0.927 (0.730–1.177)	0.533	0.865 (0.670–1.118)	0.268

Abbreviations: ALT, alanine transaminase; AST, aspartate aminotransferase; BMI, body mass index; LDL‐C, low‐density lipoprotein cholesterol; sCr, serum creatinine; ST2, suppression of tumorigenicity 2; WBC, white blood cells.

Bold values represent the correlations reached statistically significant.

### 3.4 Diagnostic potential of ST2 for ACS and NSTEMI

ROC curve analysis was performed with the laboratory indicators as the test variable, coronary CT, coronary angiography, and cTnl diagnostic results as the status variable.

**FIGURE 1:**
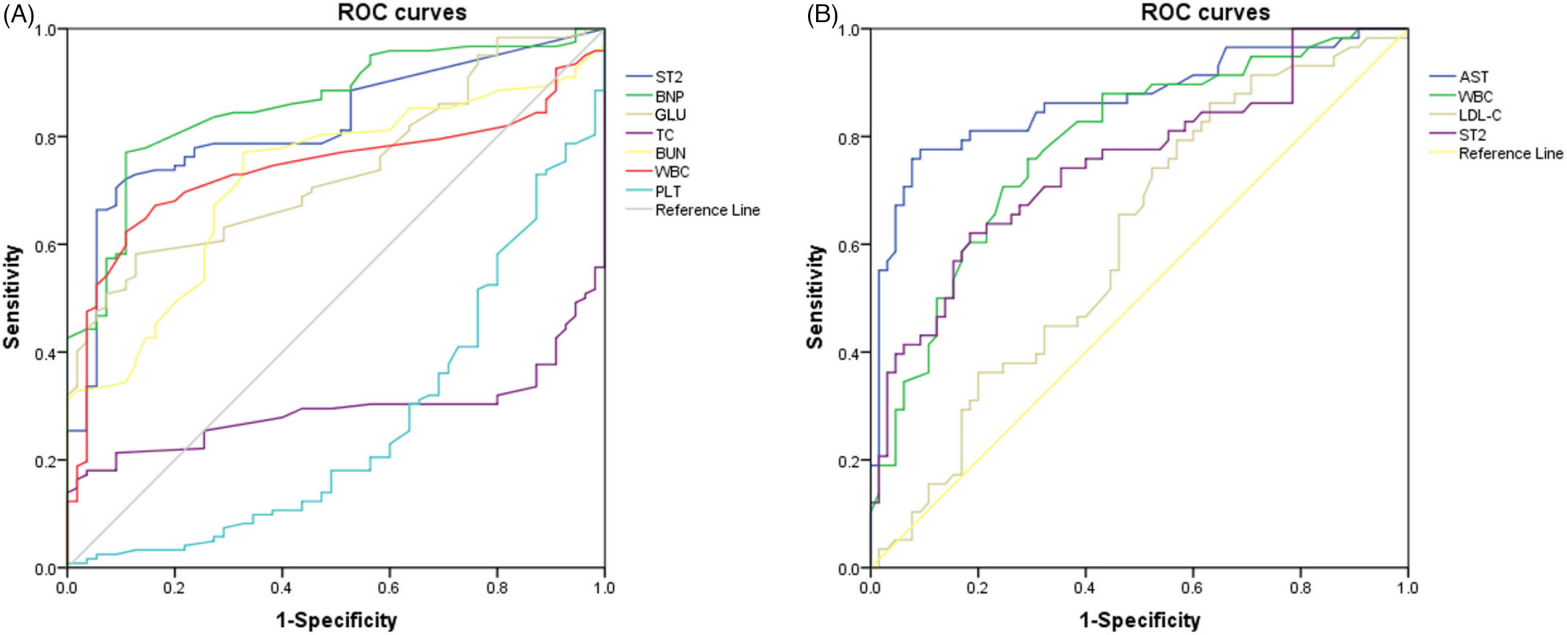
Receiver‐operating characteristic curve analysis of ST2 and other indicators on predicting in patients with ACS or NSTEMI. (A) Receiver‐operating characteristic curve analysis of ST2 and and other indicators on predicting in patients with ACS; (B) Receiver‐operating characteristic curve analysis of ST2 and and other indicators on predicting in patients with NSTEMI. AST, aspartate aminotransferase; BNP, brain natriuretic peptide; BUN, blood urea nitrogen; GLU, blood glucose; LDL‐C, low‐density lipoprotein cholesterol; PLT, platelet; ST2, suppression of tumorigenicity 2; TC, total cholesterol; WBC, white blood cells

The AUC of ST2 levels for the diagnostician of the ACS was 0.823 (95% CI, 0.761–0.886, *p* < 0.001). BNP, GLU, TC, BUN, WBC, and PLT were the other independent factors, whose AUC were 0.859 (95% CI, 0.803–0.915, *p* < 0.001), 0.739 (95% CI, 0.668–0.811, *p* < 0.001), 0.292 (95% CI, 0.217–0.367, *p* < 0.001), 0.722 (95% CI, 0.646–0.798, *p* < 0.001), 0.739 (95% CI, 0.666–0.813, *p* < 0.001), and 0.268 (95% CI, 0.187–0.349, *p* < 0.001), respectively (Table 5). In addition, our study revealed that the AUC of ST2 levels for the diagnostician of the NSTEMI was 0.748 (95% CI, 0.661–0.836, *p* < 0.001). AST, WBC and L‐DLC were the other independent factors, whose AUC were 0.869 (95% CI, 0.801–0.936, *p* < 0.001), 0.783 (95%CI, 0.701–0.864, *p* < 0.001), and 0.609 (95% CI, 0.509–0.709, *p* < 0.05), respectively (Table 6). We concluded that the diagnostic performances of ST2 for ACS were equivalent to BNP, and the diagnostic performances of ST2 for NSTEMI were equivalent to AST and WBC (Figure 1).

**TABLE 5:** Receiver‐operating characteristic curves of various biomarkers to predict ACS

Variables	Cutoff	Sensitivity	Specificity	AUC (95% CI)	*p*
ST2 (ng/ml)	7.15	0.707	0.909	0.823 (0.761–0.886)	<0.001
BNP (ng/L)	37.5	0.770	0.109	0.859 (0.803–0.915)	<0.001
GLU (mmol/L)	5.325	0.582	0.127	0.739 (0.668–0.811)	<0.001
TC (mmol/L)	7.15	0.705	0.909	0.292 (0.217–0.367)	<0.001
BUN (mmol/L)	4.35	0.770	0.673	0.722 (0.646–0.798)	<0.001
WBC (×10^9^/L)	6.05	0.623	0.891	0.739 (0.666–0.813)	<0.001
PLT (×10^9^/L)	237.5	0.205	0.600	0.268 (0.187–0.349)	<0.001

Abbreviations: BMI, body mass index; BNP, brain natriuretic peptide; BUN, blood urea nitrogen; GLU, blood glucose; PLT, platelet; ST2, suppression of tumorigenicity 2; TC, total cholesterol; WBC, white blood cells.

**TABLE 6:** Receiver‐operating characteristic curves of various biomarkers to predict NSTEMI

Variables	Cutoff	Sensitivity	Specificity	AUC (95% CI)	*p*
AST (U/L)	25.95	0.776	0.906	0.869 (0.801–0.936)	<0.001
WBC (×10^9^/L)	6.45	0.759	0.703	0.783 (0.701–0.864)	<0.001
ST2 (ng/ml)	19.6	0.621	0.812	0.748 (0.661–0.836)	<0.001
L‐DLC (mmol/L)	1.895	0.862	0.375	0.609 (0.509–0.709)	0.042

Abbreviations: AST, aspartate aminotransferase; LDL‐C, low‐density lipoprotein cholesterol; ST2, suppression of tumorigenicity 2; WBC, white blood cells.

## 4 DISCUSSION

This study assessed serum ST2 levels in the short‐term after the onset of ACS‐related symptoms in patients. Our results suggest that ST2 concentrations at the onset of chest pain may be a useful biomarker of ACS for clinical decision‐making in patients admitted to the emergency department or outpatient clinics with chest pain.

Acute coronary syndrome (ACS) is one of the major fatal and disabling diseases affecting millions of people worldwide. ACS leads to compromised diastolic function and myocardial ischemia,^17^ when cardiomyocytes and cardiac fibroblasts are subjected to injury and mechanical stress, a significant increase of release in ST2L and ST2, which acts as a decoy receptor for IL‐33 and can competitively inhibit the binding of IL‐33 to ST2L, thus limiting the protective effect of IL‐33 on the heart.^18^ This study demonstrated that the ST2 level was significantly increased in patients with ACS compared with controls. Also, we found there was a tendency that the concentrations of ST2 of patients in the NSTEMI subgroups were higher than in USAP subgroups and controls, and the concentration of ST2 was higher in the USAP subgroup than in controls, which implied that the concentration of ST2 increases with the severity of myocardial ischemia.

Platelets and the coagulation system are key factors in the initiation, amplification, and perpetuation of ACS.^19^ Inflammatory response is involved in the pathology of Cardiogenic shock (CS) and AMI.^20^ Kamińska J et al.^21^ measured PLT, MPV, LPLT, and WBC in patients with ACS and found that inflammation and platelet activation indicators may be associated with myocardial ischemia and myocardial injury. Our study found significantly higher WBC levels in the ACS group than in the control group, which was consistent with previous studies. And PLT was notably lower than in the control group. The decreased PLT count might represent increased PLT consumption at the AMI site and elsewhere due to hyperactivity.^22^ In contrast, the PLT level was equal in the USAP and NSTEMI groups, which indicated that PLT depletion was comparable in USAP and NSTEMI patients.

In addition, we found that levels of BUN were significantly higher in ACS than in controls. It may be related to the fact that the sympathetic and renin‐angiotensin systems are activated when ACS occurs, and the increase in reabsorption in the proximal renal tubule leads to an increased concentration of BUN.^23^

AST is found in large numbers in the heart muscle cells and liver. When the supplement of blood is reduced, a large amount of AST in the cytosol enters the bloodstream, resulting in a significant increase in AST levels, and therefore, AST is used as an adjunct to epicardial coronary artery disease or liver disease. Liver insufficiency is common in heart disease, elevated AST is associated with an increased risk of cardiac‐related death if no other cause of liver damage is found.^24^ In this study, we found that the levels of AST were significantly higher in NSTEMI than in USAP, however, not in the control and ACS groups. And the absence of severe liver and kidney disease in the population included in this study suggests that elevated AST is associated with myocardial necrosis in patients with NSTEMI.

Cardiac fibrosis after myocardial infarction is one of the common clinical types of myocardial fibrosis, and the core pathology is that the main effector cells of myocardial fibrosis after myocardial infarction are induced by pro‐fibrotic factors such as oxidative stress and inflammatory factors, which leads to changes in biological behaviors such as proliferation, phenotypic differentiation, migration, and secretion, resulting in massive deposition of extracellular matrix, cardiac remodeling, and fibrosis, affecting cardiac diastolic function and electrical signal. It also leads to heart failure and arrhythmias, and affects prognosis.^25^, ^26^ ST2 has been demonstrated as a marker of myocardial fibrosis.^27^ In the present study, we found that serum ST2 levels were positively correlated with ALT, AST, and BNP in the control population, and that ST2 levels were negatively correlated with HGB and TG in USAP patients, while ST2 levels were positively correlated with WBC, GLU, and BNP in NSTEMI patients, which suggested an interrelationship between these variables.

The Gensini score is a scale that reflects the degree of coronary artery stenosis in subjects and is widely used in clinical practice. In the study, we found that ST2 level was positively correlated with the Gensini score, which suggested that ST2 levels in NSTEMI patients were related to the degree of coronary artery stenosis.

In addition, we found there was a limited association between ST2 and cTnI, which suggested that the biological behavior of myocardial fibrosis and myocardial infarction are not synchronous after ACS.

ROC curve analysis shows that ST2 has good diagnostic efficacy for ACS as well as NSTEMI, even after adjusting for age, gender and BMI. When ST2 is higher than 7.15 ng/ml or 19.6 ng/ml, it means the occurrence of ACS and NSTEMI, and this was different from the cutoff value for heart failure.^14^ Interestingly, the inclusion of BMI in a multivariate logistic regression model revealed that BMI was an independent risk factor for the occurrence of ACS. It is suggested that the risk of ACS occurrence increases with increasing body mass index.

This study has some limitations. On the one hand, stable angina was not included in this study because it has been studied in many reports, and the relationship between controls and unstable angina has been clarified. On the other hand, although ST2 could reflect the degree of myocardial fibrosis and differentiate between USAP and NSTEMI, it is only used as a diagnostic indicator in the early stages of disease onset and the prognosis of patients is not yet known. Therefore, in future studies, we will continue to return to investigate the efficacy of ST2 on the prognosis of patients.

## CONFLICT OF INTEREST

The authors declare that they have no competing interests.

## Data Availability

All data generated or used during the study appear in the submitted article.
